# Outcomes in Catheter Ablation of Sustained Ventricular Tachycardia in Myocarditis Compared with Ischemic Heart Disease

**DOI:** 10.31083/RCM25604

**Published:** 2025-01-09

**Authors:** Sheng Su, Le Li, Xi Peng, Likun Zhou, Zhuxin Zhang, Yulong Xiong, Zhenhao Zhang, Mengtong Xu, Yan Yao

**Affiliations:** ^1^Arrhythmia Center, Fuwai Hospital, Chinese Academy of Medical Sciences & Peking Union Medical College, National Center for Cardiovascular Diseases, 100037 Beijing, China

**Keywords:** catheter ablation, sustained ventricular tachycardia, outcome, myocarditis, ischemic heart disease

## Abstract

**Background:**

The substrates for arrhythmias in myocarditis and ischemic heart disease (IHD) are different, but it is yet to be determined whether there is a difference in outcomes following catheter ablation (CA) for ventricular tachycardia (VT) associated with these two conditions. This study aimed to compare outcomes after CA of VT in patients with myocarditis versus those with IHD.

**Methods:**

Patients undergoing CA for sustained VT confirmed by endomyocardial biopsy as myocarditis, and patients with IHD experiencing sustained VT undergoing CA were retrospectively enrolled from February 2017 to March 2023. Initially, an endocardial approach was employed, reserving epicardial ablation procedures for non-responders. The primary endpoint was VT recurrence during follow up. All-cause mortality, repeat CA for VT and implantable cardioverter-defibrillator (ICD) implantation served as secondary endpoints. Kaplan-Meier curves compared outcomes between patient groups.

**Results:**

This study included 109 patients with IHD and 20 patients with myocarditis who underwent CA for sustained VT, from February 2017 to March 2023. Compared with IHD patients, myocarditis patients had a statistically significant lower complete short-term success rate of CA (60.0% vs. 85.3%, *p* = 0.013). During a follow-up period of 37 ± 21 months, 8 (40.0%) myocarditis patients experienced VT recurrence compared to 57 (52.3%) IHD patients, with no statistically significant difference between the two groups. During follow-up, 2 (10.0%) myocarditis patients died and 2 (10.0%) underwent repeat CA for VT recurrence, while 9 (8.3%) IHD patients died, 14 (12.8%) underwent a second CA for VT recurrence, and 8 (7.3%) received an ICD implantation. Additionally, there were no notable variations between the two groups regarding all-cause mortality, repeat CA for VT and ICD implantation.

**Conclusions:**

It was demonstrated that the efficacy of CA in sustained VT in myocarditis patients was similar to that in IHD. For myocarditis patients with VT, CA might be equally effective.

## 1. Introduction 

Myocarditis, defined as inflammatory injury of the myocardium that can involve the cardiac conduction system and pericardial layers [1], affects approximately 10 to 22 people per 100,000 per year globally [2]. Previous studies have suggested that the probability of ventricular tachycardia (VT) after myocarditis is 6% [3] and the incidence of ventricular arrhythmias is as high as 55% in some specific types of myocarditis [4]. The VT may lead to an adverse short-term prognosis and is also a common mechanism of cardiac death [5, 6].

Recent study indicates that catheter ablation (CA) for VT is both effective and safe [7]. Studies in VT ablation in ischemic heart disease (IHD) patients have shown encouraging results [8, 9, 10]. However, data on myocarditis is scarce and most studies included a mix of patients without histological validation. The recurrence rates for VT in myocarditis patients who underwent CA range from 23–34% [11, 12]. The VT in myocarditis relates to inflammation, while that in IHD is linked to scarring [13, 14]. The arrhythmia substrates vary between these conditions. Therefore, there may be differences in ablation outcomes. However, fewer studies have compared CA outcomes for VT in myocarditis patients versus those with IHD. This study was conducted retrospectively to compare the outcomes after CA of VT in patients with myocarditis confirmed by histological validation and IHD.

## 2. Materials and Methods

### 2.1 Study Population

This is a single-center retrospective study. This study consecutively enrolled myocarditis patients confirmed by endomyocardial biopsy (EMB) who underwent CA for sustained VT at the Fuwai Hospital, Chinese Academy of Medical Sciences from February 2017 to March 2023. Patients with IHD undergoing CA for sustained VT during the same period were included for comparison. Clinical presentations, family history, comorbidities, 12-lead electrocardiogram (ECG) results, and EMB of all patients were obtained from the electronic medical record system.

Sustained VT was defined as either lasting more than 30 seconds or necessitating termination within 30 seconds due to hemodynamic compromise [15]. The diagnosis of myocarditis was based on the pathological criteria of the current guideline [16]. EMB was performed via the right femoral vein to obtain myocardial tissue from the right ventricular septum. Myocarditis was pathologically diagnosed and staged according to the Dallas criteria [17]. In general, acute myocarditis showed myocardial cell necrosis and inflammation activation, while chronic myocarditis involved both destruction and remodeling. For patients with myocarditis, EMB was performed concurrently with CA using Jawz 2.2 mm Forceps, Maxi-Curved, 105 cm (Argon Medical Devices, Frisco, TX, USA). IHD was a cardiovascular condition characterized by diminished myocardial blood flow resulting from coronary artery disease [18].

### 2.2 CA Procedure

Prior to the ablation procedure, all patients provided informed consent and were prepared following the standard clinical protocol of our department. Mapping and CA procedures were carried out under local anesthesia and sedation. ECG monitoring was conducted continuously throughout the procedure. A decapolar steerable electrode catheter was introduced into the coronary sinus through femoral venous access, while a standard fixed-curve quadripolar catheter was positioned in the right ventricle. Ventricular programmed or incremental stimulation was used to induce clinical VT until the ventricular refractory period was reached or VT onset occurred. For inducible and tolerable VTs, activation mapping and entrainment mapping were performed to identify critical isthmuses of reentry; for uninducible or unstable VTs, substrate mapping under sinus rhythm were performed. Electroanatomic mapping was conducted using either the CARTO 3D electroanatomical mapping system (Biosense Webster, Diamond Bar, CA, USA) or the Ensite Precision 3D electroanatomical mapping system (Abbott Laboratories, St. Paul, MN, USA). Endocardial mapping-guided ablation was performed in all patients. For those in whom endocardial ablation failed, epicardial mapping via subxiphoid pericardial puncture was considered. The arrhythmogenic substrates comprised split electrograms, low voltage (≤0.5 mV), or fractionated electrograms, which were characterized by multiple potentials with ≥2 distinct components, >20 ms of isoelectric segments between the peaks of these components, and either a long duration (>80 ms) or late potentials.

Ablation was conducted with radiofrequency energy, set at a target temperature of 45 °C and a maximum power of 50 W, utilizing irrigation at a flow rate of 12–30 cc/min. The ablation catheters used for catheter ablation were the Flexibility^TM^ ablation catheter (Abbott Laboratories, St. Paul, MN, USA) or the THERMOCOOL SMARTTOUCH ablation catheter (Biosense Webster, Diamond Bar, CA, USA). For ablations guided by activation mapping, the effectiveness of the ablation is evaluated post-procedure by repeating ventricular stimulation. If no VT can be induced, the ablation is defined as successful. If clinical VT cannot be induced, but other VT morphologies are inducible, the ablation is considered partially successful. If clinical VT is still inducible, the ablation is deemed a failure. For ablation guided by substrate mapping, the procedure target was eliminating all arrhythmogenic substrates.

### 2.3 Follow-up and Outcomes

Patients were followed up through phone calls or clinic visits at 3, 6, and 12 months after discharge, and then annually thereafter. Regular telephone interviews were conducted with patients or their family members as well. At each follow-up, patients underwent 12-lead ECG and 24-hour Holter monitoring to identify arrhythmias. For those with an implantable cardioverter-defibrillator (ICD), device checks were performed every 6 months. The primary endpoint of this study was recurrent VT. VT recurrence was defined as sustained VT (duration >30 s), documented by ECG or Holter monitoring, or appropriate ICD shocks. During the follow-up period, occurrences such as all-cause mortality, repeat CA for VT and ICD implantation would also be documented. Every effort was made to ascertain the causes of death for the patients.

### 2.4 Statistical Analysis

The normality of the data was assessed using the Kolmogorov–Smirnov test. Continuous variables were expressed as means ± standard deviations or as medians with interquartile ranges (25th–75th percentiles), depending on the data distribution. Comparisons were performed using the *t*-test for normally distributed data and the Mann–Whitney U test for non-normally distributed data. Categorical variables were presented as counts and percentages, and comparisons were made using the χ^2^ test or Fisher’s exact test. To enhance credibility, propensity score matching (PSM) was employed to reduce potential confounders and selection bias in this retrospective study. Propensity scores were computed based on the characteristics outlined in Table 1. One-to-two nearest-neighbor matching was performed using a 0.25 caliper. After matching, a total of 32 patients were obtained. Standardized mean differences (SMD) were used to assess the differences between the matched groups, with a maximum SMD of 0.1 or even 0.15 was typically considered acceptable.

**Table 1.** **Baseline characteristics**.

		Unmatched				Matched			
		IHD	Myocarditis	*p*	SMD	IHD	Myocarditis	*p*	SMD
		n = 109	n = 20			n = 20	n = 12		
Male		101 (92.7%)	13 (65.0%)	0.002	0.72	16 (80.0%)	8 (66.7%)	0.673	0.305
Age, years		60 ± 10	43 ± 12	<0.001	1.59	50 ± 11	49 ± 11	0.743	0.12
Hypertension		60 (55.0%)	4 (20.0%)	0.008	0.776	10 (50.0%)	3 (25%)	0.307	0.535
Diabetes		29 (26.6%)	0	0.02	0.851	6 (30.0%)	0	0.102	0.926
CKD		6 (5.5%)	2 (10.0%)	0.793	0.169	1 (5.0%)	2 (16.7%)	0.639	0.382
ICD history		29 (26.6%)	5 (25.0%)	1	0.037	2 (10.0%)	4 (33.3%)	0.242	0.591
CA history		14 (12.8%)	3 (15.0%)	1	0.062	3 (15.0%)	2 (16.7%)	1	0.046
Smoking		67 (61.5%)	6 (30.0%)	0.018	0.666	11 (55.0%)	4 (33.3%)	0.41	0.447
Drinking		43 (39.4%)	3 (15.0%)	0.065	0.571	6 (30.0%)	3 (25.0%)	1	0.112
LVEF, %		48 ± 11	54 ± 12	0.016	0.559	52 ± 8	53 ± 13	0.883	0.051
NYHA III/IV		14 (12.8%)	3 (15.0%)	1	0.062	0	1 (8.3%)	0.793	0.426
VT with CHD		98 (89.9%)	17 (85.0%)	0.797	0.149	17 (85.0%)	10 (83.3%)	1	0.046
Medicine									
	Amiodarone	61 (56.0%)	12 (60.0%)	0.929	0.082	8 (40.0%)	6 (50.0%)	0.854	0.202
	β-blocker	89 (81.7%)	16 (80.0%)	1	0.042	15 (75.0%)	9 (75.0%)	1	<0.001

CA, catheter ablation; CHD, compromised hemodynamics; CKD, chronic kidney disease; ICD, implantable cardioverter defibrillator; IHD, ischemic heart disease; LVEF, left ventricular ejection fraction; NHYA, New York Heart Association; VT, ventricular tachycardia; SMD, standardized mean differences.

Event-free survival was assessed using the Kaplan–Meier method and analyzed with the log-rank test. Cox proportional hazard modeling was then performed, incorporating potential confounders identified from significant univariate associations (*p* < 0.05). Multivariable Cox regression analysis was conducted to determine significant predictors of VT recurrence, accounting for relevant clinical covariates. Statistical analyses were performed using R software version 4.1.0 (R Foundation for Statistical Computing, Vienna, Austria). All tests were two-tailed, with statistical significance defined as *p* < 0.05.

## 3. Results

### 3.1 Baseline Characteristics

**Fig. 1.**
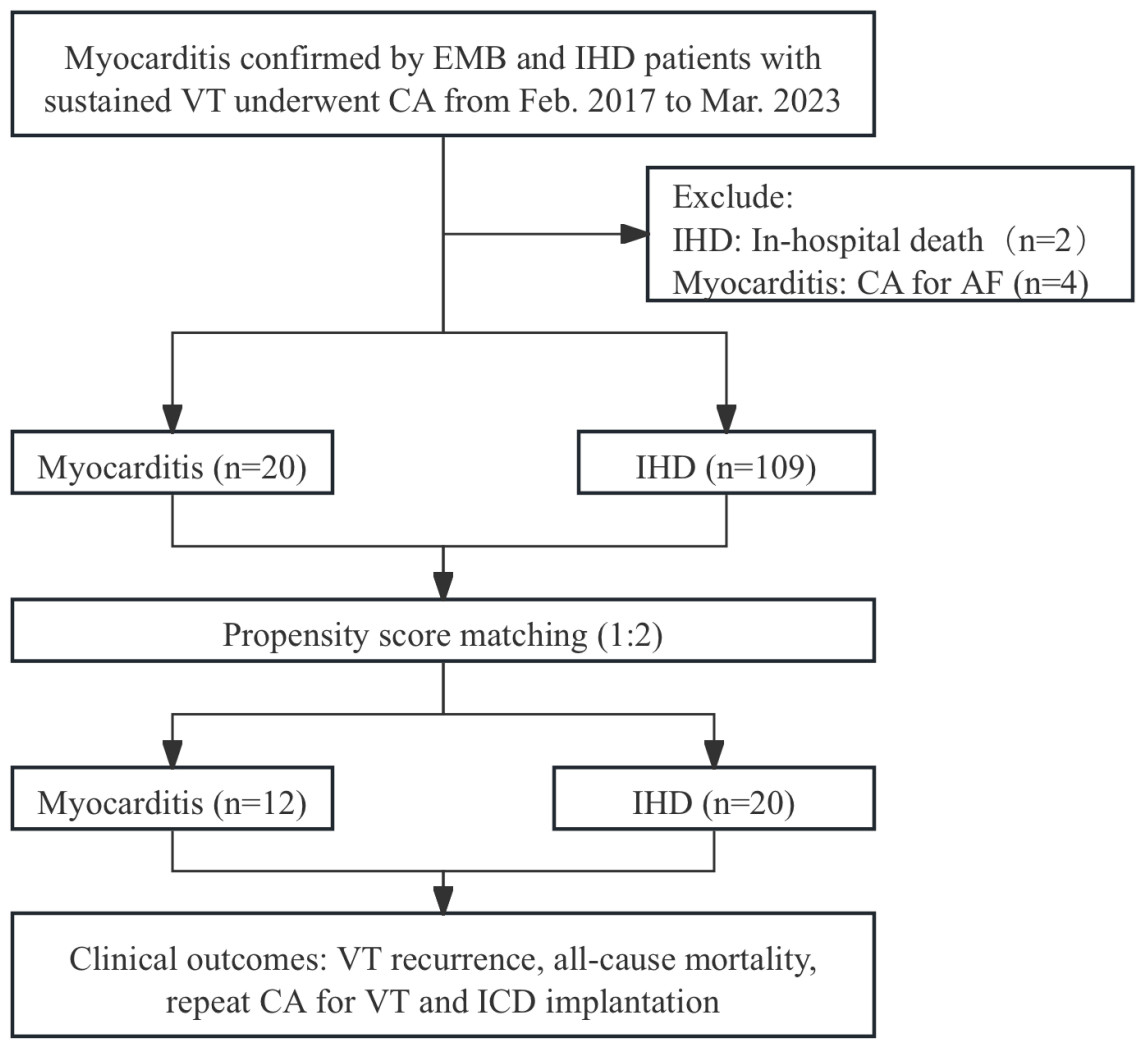
**Study flowchart**. AF, atrial fibrillation; CA, catheter ablation; EMB, endomyocardial biopsy; ICD, implantable cardioverter-defibrillator; IHD, ischemic heart disease; VT, ventricular tachycardia.

During the study period, 109 IHD patients and 20 myocarditis patients with proven EMB were enrolled (Fig. 1). The mean age was 57 ± 12 years old and the mean left ventricular ejection fraction (LVEF) was 48 ± 11%. 114 (88.4%) patients were male. 115 (89.1%) patients had a history of requiring termination of VT episodes due to compromised hemodynamics. In this population, the comorbidities ranked from high to low were hypertension (49.6%), diabetes (22.5%), and chronic kidney disease (6.2%). 73 (56.6%) patients had a history of smoking and 46 (35.7%) had a history of drinking. In addition, 34 (26.4%) patients had a history of ICD implantation and 17 (13.2%) had a history of CA. The baseline characteristics of the patient population are summarized in Table 1. Compared to IHD patients enrolled, patients with myocarditis have a significantly higher proportion of females, younger age, fewer comorbidities of hypertension, diabetes, smoking, and better LVEF (Table 1). After matching, no statistical differences were found between the 2 groups in all covariates (Table 1). In the myocarditis cohort, 15 (75.0%) patients were in the chronic stage while 5 (25.0%) patients were in the acute stage based on the results of the pathology report. All patients with myocarditis exhibited resistance to anti-arrhythmic drug therapy. There was no significant difference in the history of requiring termination of VT episodes due to compromised hemodynamics between acute and chronic myocarditis. In addition, 4 patients had giant cell myocarditis, 9 patients had lymphocytic myocarditis, 6 patients had atypical myocarditis, and 1 patient had cardiac sarcoidosis-related myocarditis. 10 patients received immunosuppressive therapy combined with steroid treatment, 3 patients received steroid treatment and the remaining 7 patients refused to undergo steroid or immunosuppressive therapy.

### 3.2 Electrophysiological Findings in the Procedures

**Fig. 2.**
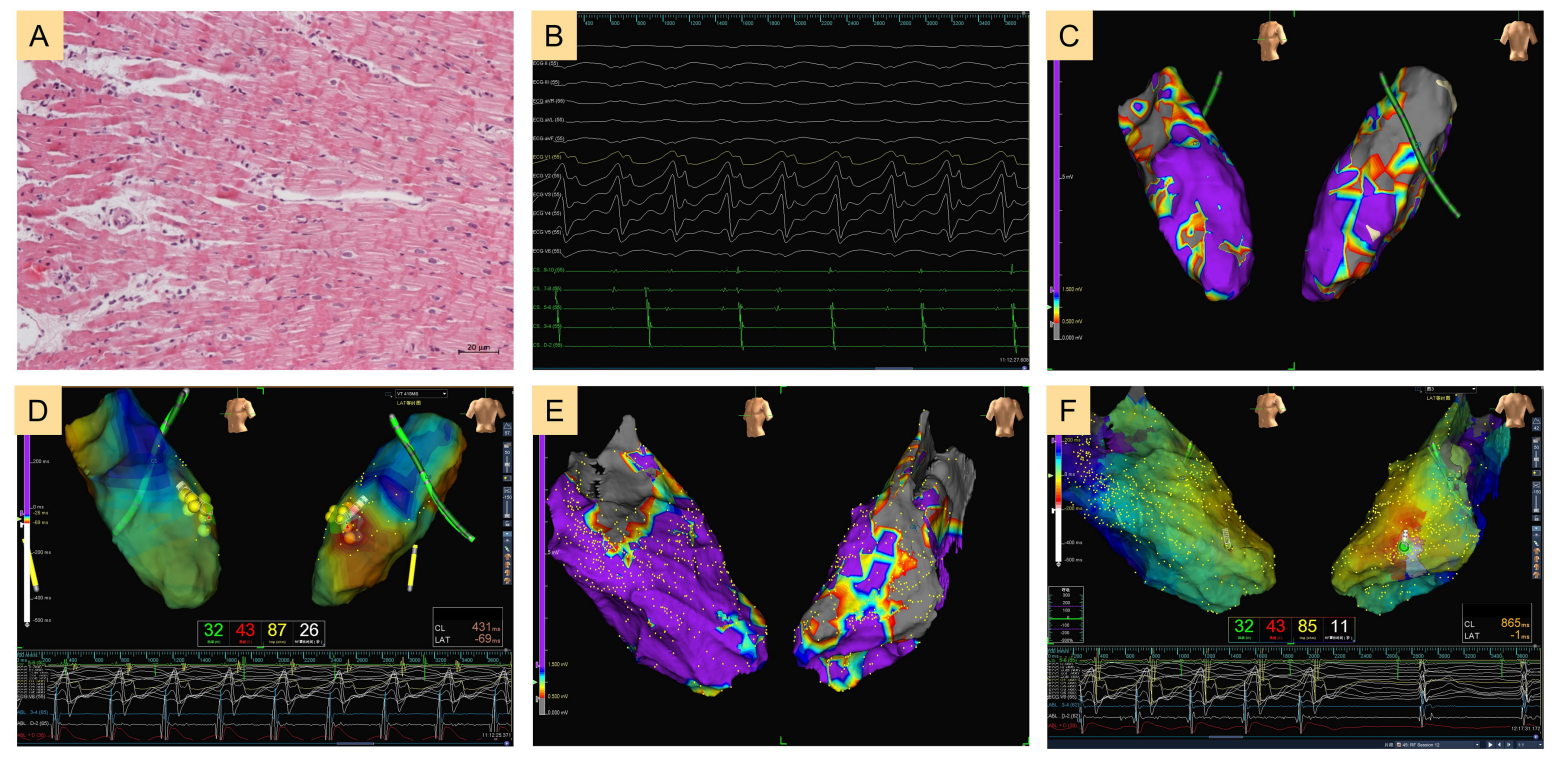
**Sustained ventricular tachycardia in a patient with myocarditis**. (A) The endomyocardial biopsy finding. Scale bar: 20 µm. (B) The electrocardiogram of sustained ventricular tachycardia. (C) Left ventricular endocardial substrate mapping results. (D) Activation mapping results and failure of ventricular tachycardia ablation. (E) Left ventricular epicardial substrate mapping results. (F) Activation mapping results and success of ventricular tachycardia ablation. ABL, ablation; CL, cycle length; LAT, local activation time; ECG, electrocardiogram; CS, coronary sinus.

**Fig. 3.**
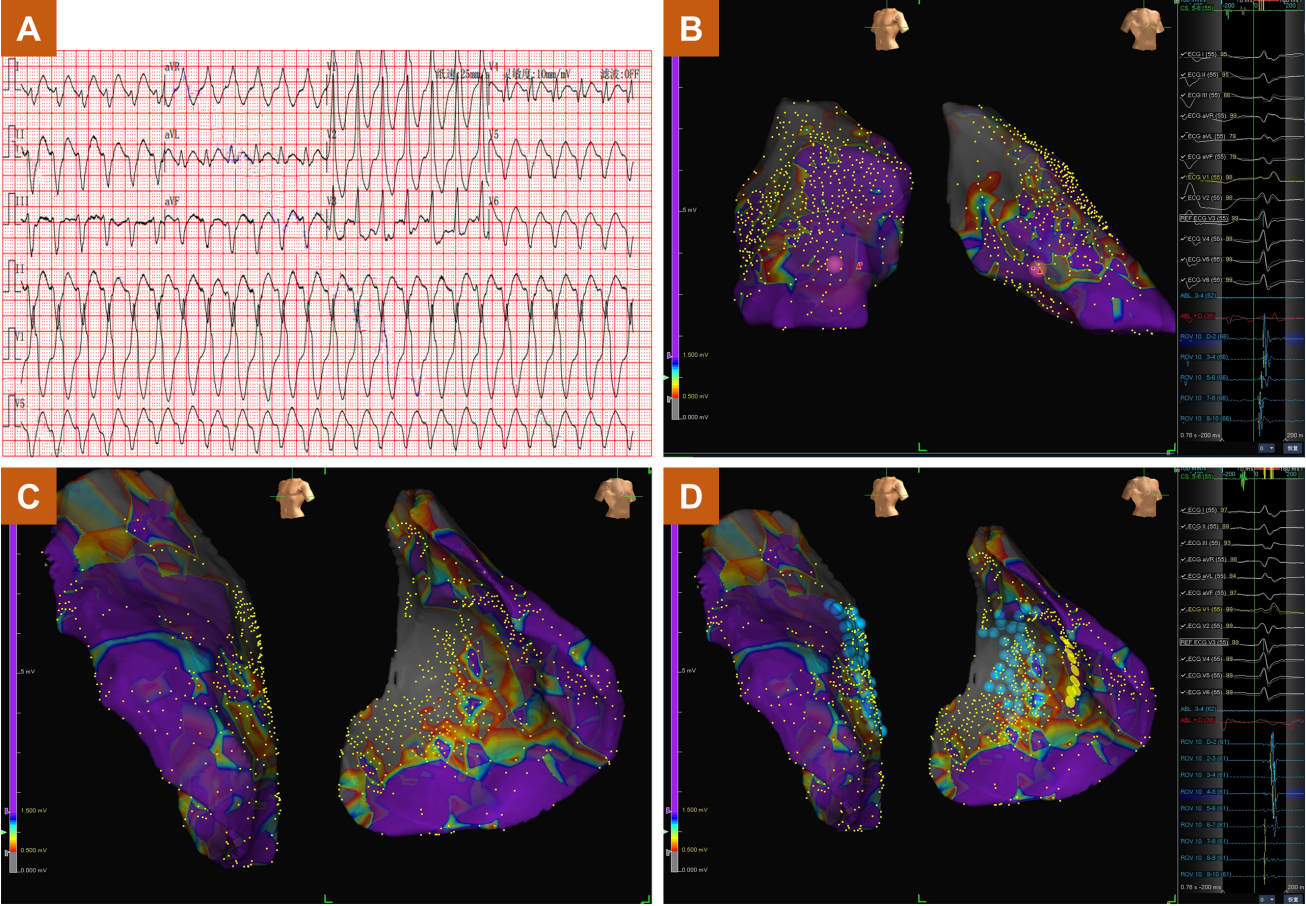
**Sustained ventricular tachycardia in a patient with ischemic heart disease**. (A) The electrocardiogram of sustained ventricular tachycardia. (B) The endocardial substrate mapping results. (C) The epicardial substrate mapping results. (D) Successful ablation based on epicardial delayed potentials. ABL, ablation; ECG, electrocardiogram; REF, reference electrode; CS, coronary sinus.

In this study, activation mapping was performed in 21 (16.3 %) patients and substrate mapping was performed in 100 (77.5%) patients. 8 (6.2%) patients underwent epicardial mapping due to failed endocardial ablation. During the procedures, 20 (15.5%) patients experienced hemodynamically unstable VT requiring defibrillation. Table 2 presented the electrophysiological findings and the specific origin of VT was in **Supplementary Table 1**. Out of the 129 patients, 105 (81.4%) achieved complete success with CA, 20 (15.5%) experienced partial success, and 4 (3.1%) encountered failure. 121 (93.8%) patients underwent endocardial CA and 8 (6.2%) patients underwent epicardial CA. Figs. 2,3 show the cardiac electrophysiological findings of a patient with myocarditis and a patient with IHD, respectively. In terms of CA complications, 3 patients developed pericardial effusion, with 1 requiring pericardial drainage. During hospitalization, 13 (10.1%) patients underwent ICD implantation following CA. Patients declined ICD implantation primarily because of economic concerns, but also out of fear of potential complications.

**Table 2.** **Electrophysiological findings in the procedures**.

		IHD	Myocarditis	*p*	IHD	Myocarditis	*p*
		n = 109	n = 20		n = 20	n = 12	
Activation mapping, n (%)		9 (8.3%)	12 (60.0%)	<0.001	3 (15.0%)	7 (58.3%)	0.002
Substrate mapping, n (%)		95 (87.2%)	5 (25.0%)	<0.001	15 (75.0%)	3 (25.0%)	0.006
Epicardial mapping, n (%)		5 (4.6%)	3 (15.0%)	0.107	2 (10.0%)	2 (16.7%)	0.620
VTs in the procedure							
	1	77 (70.6%)	9 (45.0%)	0.038	16 (80.0%)	6 (50.0%)	0.119
	2	14 (12.8%)	5 (25.0%)	0.174	2 (10.0%)	3 (25.0%)	0.338
	≥3	18 (16.5%)	6 (30.0%)	0.208	2 (10.0%)	3 (25.0%)	0.338
VT CL, ms		334 ± 56	331 ± 36	0.682	321 ± 46	340 ± 20	0.156
VT origin							
	Left ventricular	99 (90.8%)	8 (40.0%)	<0.001	16 (80.0%)	5 (41.7%)	0.053
	Right ventricular	5 (4.6%)	9 (45.0%)	<0.001	2 (10.0%)	5 (41.7%)	0.073
	Epicardial origin	5 (4.6%)	3 (15.0%)	0.107	2 (10.0%)	2 (16.7%)	0.620
Defibrillation		19 (17.4%)	1 (5.0%)	0.120	3 (15.0%)	0	0.274

IHD, ischemic heart disease; VT, ventricular tachycardia; VT CL, ventricular tachycardia cycle length.

Patients with myocarditis more commonly underwent activation mapping and less frequently undergo substrate mapping, compared with IHD patients (Table 2). Of note, myocarditis patients had a significantly lower rate of complete success than IHD patients [12 (60.0%) vs. 93 (85.3%), *p* = 0.013] (Table 2). However, the CA failure rates of the two groups of patients did not show a statistically significant difference. There was no notable contrast in terms of CA complications and ICD implantation when comparing patients with IHD to those with myocarditis (all *p* > 0.05). The CA outcomes of the matched cohorts were consistent with the results described above (Table 3). In the myocarditis cohort, the overall complete success rate of CA during the acute myocarditis was 40.0%, which appeared to be lower than that observed in the chronic stage (66.7%), although this difference did not reach statistical significance (*p* = 0.347). The complete success rate of CA in patients with chronic myocarditis are comparable to that in IHD patients [10 (66.7%) vs. 93 (85.3%), *p* = 0.132] and was significantly lower in acute myocarditis patients than that in IHD patients [2 (40.0%) vs. 93 (85.3%), *p* = 0.032]. One patient in each of the acute myocarditis subgroup and chronic myocarditis subgroup developed pericardial effusion after CA, with no statistical difference observed. One patient in the acute myocarditis subgroup underwent ICD implantation following CA.

**Table 3.** **In-hospital and follow-up outcomes**.

			IHD	Myocarditis	*p*	IHD	Myocarditis	*p*
			n = 109	n = 20		n = 20	n = 12	
In-hospital								
	CA result							
		failure	4 (3.7%)	0	1	0	0	-
		partial success	12 (11.0%)	8 (40.0%)	0.003	1 (5.0%)	4 (33.3%)	0.053
		complete success	93 (85.3%)	12 (60.0%)	0.013	19 (95.0%)	8 (66.7%)	0.053
	CA complication, n (%)		1 (0.9%)	2 (10.0%)	0.095	0	1 (8.3%)	0.793
	ICD implantation, n (%)		12 (11.0%)	1 (5.0%)	0.677	1 (5.0%)	1 (8.3%)	1
Follow-up								
	VT recurrence		57 (52.3%)	8 (40.0%)	0.843	15 (75.0%)	5 (41.7%)	0.515
	Re-CA for VT recurrence		14 (12.8%)	2 (10.0%)	0.799	8 (40.0%)	1 (8.3%)	0.257
	Death		9 (8.3%)	2 (10.0%)	0.188	0	1 (8.3%)	0.157
	Cardiac death		7 (6.4%)	2 (10.0%)	0.145	0	1 (8.3%)	0.157
	Anti-tachycardia pacing		21 (19.3%)	0	0.115	3 (15.0%)	0	0.248
	ICD implantation		8 (7.3%)	0	0.312	3 (15.0%)	0	0.281
	LVEF^*^, %		47 ± 10	54 ± 11	0.077	55 ± 8	51 ± 12	0.449
	LVEF change^*^, %		0 (–2, 3)	2 (–1, 7)	0.532	0 (–2, 2)	0 (–1, 7)	0.586

CA, catheter ablation; ICD, implantable cardioverter defibrillator; IHD, ischemic heart disease; LVEF, left ventricular ejection fraction; Re-CA, repeat-catheter ablation; VT, ventricular tachycardia. ^*^, 93 patients had transthoracic echocardiography data during the follow-up.

### 3.3 Follow-up Outcomes

**Fig. 4.**
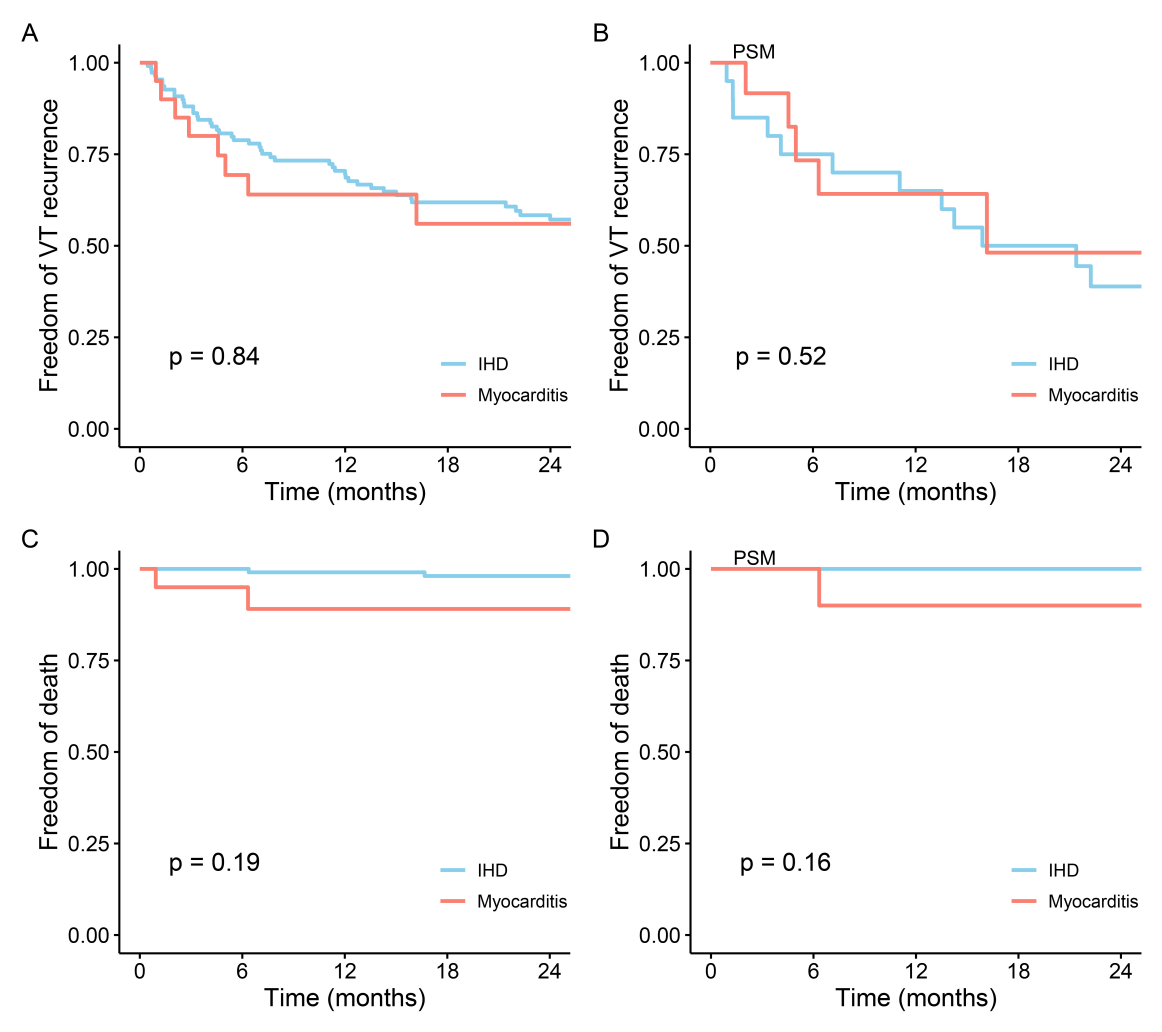
**Comparison of follow-up outcomes after catheter ablation for IHD and myocarditis**. (A) Comparison of recurrence of VT after catheter ablation in IHD and myocarditis among all patients. (B) Comparison of recurrence of VT after catheter ablation in IHD and myocarditis in the PSM cohort. (C) Comparison of death after catheter ablation in IHD and myocarditis among all patients. (D) Comparison of death after catheter ablation in IHD and myocarditis in PSM cohort. IHD, ischemic heart disease; VT, ventricular tachycardia; PSM, propensity score matching.

In a follow-up with an average duration of 37 ± 21 months, 65 (50.3%) patients experienced a recurrence of VT, 21 (16.3%) underwent ICD discharge, 16 (12.4%) patients underwent a second CA for VT recurrence, 8 (6.2%) patients received an ICD and 11 (8.5%) patients died. Among them, 9 (7.0%) patients died from cardiovascular causes. In the myocarditis cohort, 8 (40.0%) patients experienced a recurrence of VT, 2 (10.0%) patients underwent a second CA, and 2 (10.0%) died (Table 3). In addition, no patient underwent ICD implantation or experienced anti-tachycardia pacing during follow up in the myocarditis cohort (Table 3). In the IHD cohort, 57 (52.3%) patients experienced a recurrence of VT, 14 (12.8%) patients underwent a second CA for VT recurrence, and 9 (8.3%) died (Table 3). The one-year and two-year estimated rates of VT recurrence freedom in the myocarditis cohort were 64.0% (95% CI: 45.8%–98.4%) and 56.0% (95% CI: 36.6%–85.7%), respectively. The estimated freedom from death in the myocarditis cohort at one and two years was 89.1% (95% CI: 75.8%–100%) for both times. There was no statistically significant difference between the two groups in the aforementioned outcome events (Fig. 4). Similar follow up outcomes were also identified within the matched cohorts (Fig. 4). 93 patients had transthoracic echocardiography data during the follow-up period and the last LVEF was 47 ± 11%. There was no statistically significant difference between the final LVEF and the baseline LVEF (48 ± 11% vs. 47 ± 11%, *p* = 0.138) and similar conclusion was also found in the myocarditis subgroup and the IHD subgroup (both *p* > 0.05). Multivariable Cox regression analysis indicated that being male and having a longer VT cycle length were protective factors for VT recurrence, while a history of ICD implantation was identified as a risk factor (Table 4).

**Table 4.** **Multivariable Cox regression analysis for the VT recurrence**.

		Univariate		Multivariate	
		HR (95% CI)	*p*	HR (95% CI)	*p*
Female		0.487 (0.246, 0.965)	0.039	0.498 (0.252, 0.985)	0.045
NYHA III/IV		1.253 (0.636, 2.468)	0.515	-	0.931
Myocarditis		1.078 (0.510, 2.278)	0.844	-	0.774
CA outcomes					
	Failure vs. complete success	1.202 (0.291, 4.961)	0.799	-	0.990
	Partial vs. complete success	0.666 (0.314, 1.412)	0.289	-	0.143
Length of VT, ms		0.995 (0.990, 1.000)	0.056	0.995 (0.990, 1.000)	0.034
Number of VT		1.111 (0.936, 1.320)	0.228	-	0.952
ICD history		1.655 (0.977, 2.804)	0.061	1.769 (1.042, 3.004)	0.035
CA history		1.368 (0.671, 2.788)	0.388	-	0.340

CA, catheter ablation; CI, confidence interval; HR, hazard ratio; ICD, implantable cardioverter defibrillator; NHYA, New York Heart Association; VT, ventricular tachycardia.

In the myocarditis cohort, 1 patient in each of the acute phase subgroup and chronic phase subgroup suffered death, with no statistical difference observed (*p* = 0.335). In the chronic myocarditis, 5 patients (33.3%) experienced VT recurrence compared to 60.0% VT recurrence in the acute myocarditis, although without statistical significance (*p* = 0.165). In addition, the recurrence rates of VT in acute myocarditis and chronic myocarditis showed no statistically significant difference compared to IHD (both *p* > 0.05). Furthermore, a single patient in the acute myocarditis underwent a second CA for VT recurrence during follow-up, while 1 chronic myocarditis patients underwent a second CA, with no statistically significant difference observed (*p* = 0.369). Of note, there was no significant difference in VT recurrence, a second CA for VT recurrence and death between patients receiving myocarditis treatment and patients not receiving (all *p* > 0.05).

## 4. Discussion

There is limited data on the short- and long-term outcomes of VT ablation in myocarditis compared to IHD. This retrospective study compared VT catheter ablation outcomes in EBM-identified myocarditis and IHD patients and revealed similar results for both conditions. In addition, being male and having a longer VT cycle length were protective factors against VT recurrence.

Myocarditis is defined as inflammatory injury of the myocardium that can involve the cardiac conduction system and pericardial layers, and is generally mild and self-limited [16, 19]. However, patients can develop a temporary or permanent impairment of cardiac function including acute cardiomyopathy with hemodynamic compromise or severe arrhythmias. Ventricular arrhythmias were associated with sudden cardiac death [20]. According to current guidelines, patients diagnosed with myocarditis and experiencing VT may be considered for implantation of an ICD [15]. Complications associated with ICD implantation and inappropriate discharges should not be overlooked. Appropriate shocks result in discomfort, diminish quality of life, shorten device lifespan, and potentially elevate mortality rates [21]. Furthermore, many individuals were unable to undergo ICD implantation due to economic reasons [22, 23]. Catheter ablation is increasingly recognized as an effective treatment option for such arrhythmias, despite limited data on ventricular arrhythmias in the context of myocarditis [24].

In patients with myocarditis, the EMB showed an inflammatory infiltrate, along with necrosis or degeneration of neighboring myocytes [17]. The immune response may lead to electrophysiological or structural changes, causing abnormalities in action potential conduction or repolarization, thereby promoting the development of arrhythmias. The arrhythmogenic substrate of IHD is typically scar-related and commonly tends to be subendocardial (thus readily accessible for ablation) [7]. In myocarditis, the arrhythmogenic substrate is commonly found in an anteroseptal or inferolateral pattern, frequently affecting perivalvular, intramural, or epicardial areas, and the coronary arteries are typically patent [7]. In this study, the CA outcomes for both groups were similar, despite their different mechanisms of arrhythmogenic substrate formation. It has been reported that CA of VT in patients with non-ischemic cardiomyopathy (NICM) has been reported to have less favorable outcomes and higher VT recurrence rates as compared to IHD patients [25]. Previous study reported that myocarditis had superior outcomes than other kinds of NICM after adjusting for potential covariates [26]. In this study, there was no statistically significant difference observed in clinical outcomes after CA between the IHD cohort and the myocarditis cohort. For myocarditis patients with drug-refractory VT, CA is equally effective. Further research is needed to understand the specific mechanism.

In this study, complete elimination of any VT was achieved in 60.0% of myocarditis patients compared with 84.5% of the IHD patients. The complete short-term success rate of CA in myocarditis was lower than that in IHD patients. Of note, the success rate of CA in patients with chronic myocarditis was comparable to that in IHD patients, which hinted that acute myocarditis patients might be with a more complex arrhythmogenic substrate. Peretto Giovanni *et al*. [12] found that CA in the acute phase was a risk factor for early VT recurrence through an observational study. In this study, the short-term success rate following VT ablation in acute myocarditis was lower compared to chronic cases, and the recurrence of VT in acute myocarditis was higher than in chronic cases, although these differences did not reach statistical significance. For myocarditis patients with concomitant VT, a delayed CA strategy could be considered if clinically feasible. It is noteworthy that the follow-up outcomes for partial success and complete success were similar, with no statistically significant difference. Although pursuing complete elimination of all inducible VTs was desirable, ablation of the clinical VT only might be acceptable when achieving complete success was challenging. No significant difference in CA failure was observed between the two groups. These findings align with earlier studies and demonstrate relatively high immediate success rates for both myocarditis and IHD [24, 26].

Myocarditis patients had a significantly lower rate of substrate mapping, such as low voltage region, delay potential distribution and fragmentation potential distribution. The short-term success rates among different mapping methods were indistinguishable in the overall cohort and in myocarditis patients. No research study or meta-analysis has demonstrated superior outcomes with the conventional approach when contrasted with substrate-based ablation. For patients who failed endocardial ablation, equivalent therapeutic effects could be achieved through epicardial ablation. Therefore, a more sophisticated ablation strategy that integrates substrate mapping with reentry circuit characterization through activation mapping should be employed in myocarditis to improve short-term success rates. Our findings support the idea of considering epicardial ablation as a subsequent step if VT remains inducible following endocardial ablation in patients with myocarditis. Given the increased risk of complications associated with epicardial ablation, this approach may be more judicious than a mandatory combined endocardial and epicardial strategy, as recommended by some authors.

At long-term follow-up after CA, 47.8% of IHD patients and 60% of myocarditis patients were free from VT and the majority of VT recurrence occurred within one year after CA. This outcome aligns with already published data in the biggest multicenter trial [24]. In the Multicenter Thermocool Ventricular Tachycardia Ablation trial, the reported VT recurrence was 47% at 6 months [27]. Arenal Ángel *et al*. [10] found that CA decreased the composite endpoint of cardiovascular death, appropriate ICD shock, hospitalization for heart failure, or severe treatment-related complications compared to antiarrhythmic drugs (AADs). In this study, although a high rate of VT recurrence was observed, no significant association was found between VT recurrence and mortality. Not every VT recurrence was lethal. Furthermore, the necessity of repeat ablation and ICD implantation for patients experiencing VT recurrence may not be immediate. This may be related to the relatively good LVEF of the study population.

In this study, female gender was an independent risk factor for recurrent VT after CA during follow-up. Distinct variations exist between women and men in the manifestation, etiology, and therapeutic response to specific arrhythmias. An international multicenter study suggested that women with structural heart disease exhibit poorer VT-free survival post-ablation compared to men, despite presenting more favorable baseline characteristics such as younger age, higher LVEF, lower incidence of VT storm, and fewer medical comorbidities [28]. In this study, the poorer prognosis seen in women might be attributed to a more complex arrhythmia substrate, because women had a higher number of VTs than men (2.1 ± 1.5 vs. 1.7 ± 1.3, *p* = 0.490) and had a higher proportion of epicardial ablation [4 (26.7%) vs. 4 (6.2%), *p* = 0.006]. This requires further in-depth research for confirmation. In this study, history of a previous CA procedure appeared to have no impact on the recurrence of VT, suggesting that the arrhythmia substrate may be dynamically changing.

It is important to acknowledge that while VT ablation can modify the existing substrate temporarily, it may not prevent the ongoing progression of the underlying disease or the development of new substrate and triggers over time. The idea of a “fixed” morphological substrate may hold true in the context of post-infarction cardiomyopathy, but in cases of myocarditis, there are unidentified factors that contribute to the evolution and alteration of the arrhythmia substrate over time. Utilizing CA and AADs remains a crucial strategy to reduce the occurrence of VT and improve clinical symptoms in patients with myocarditis and VT. Not every patient with myocarditis and concurrent VT may require an ICD, and strict patient selection criteria are needed for ICD implantation. For myocarditis patients who already have an ICD, the indication for ICD removal can be assessed based on the occurrence of VT and ICD discharges during follow-up. Epicardial biomaterials, as a potential therapeutic approach, may also play a role in the treatment of VT in patients with myocarditis in the future [29].

There exists limitations. Firstly, this study is a single-center, retrospective, nonrandomized study. In addition, the sample size included in this study was relatively small, and the follow-up period of 37 months was relatively short. Secondly, this study represents a population in the earlier stages of left ventricular remodeling and impaired systolic function. Consequently, the results for patients receiving ablation therapy at advanced stages of the disease may differ from those reported in this study. The influence of the stimulation site on scar localization was not considered. The inability to induce VT with programmed stimulation both at the start and end of the ablation procedure in certain patients creates uncertainty around the definition of short-term success in these cases and may affect the overall short-term outcomes.

## 5. Conclusions

Although the short-term success rates after VT ablation in myocarditis was significantly lower than that in IDH, the follow-up outcomes were similar. Less substrate mapping and more epicardial mapping was performed in myocarditis patients. For myocarditis patients with VT, CA might be equally effective.

## Availability of Data and Materials

The datasets used during the current study are available from the corresponding author on reasonable request.

## Supplementary Material

Supplementary material associated with this article can be found, in the online version, at https://doi.org/10.31083/RCM25604.
