# Supplementary material for: Outcomes in Catheter Ablation of Sustained Ventricular Tachycardia in Myocarditis Compared with Ischemic Heart Disease
Source: Rev Cardiovasc Med. 2025 Jan 9;26(1):25604. doi: 10.31083/RCM25604 (PMC11759966; doi:10.31083/RCM25604)
Supplement: Supplementary file 1 [file 2153-8174-26-1-25604-s1.docx]

Supplementary table 1. VT origin.

|  | Unmatched | | Matched | |
| --- | --- | --- | --- | --- |
|  | ICM (n=109) | Myocarditis (n=20) | ICM (n=20) | Myocarditis (n=12) |
| Left ventricular |  |  |  |  |
| Free wall | 12 (11.0%) | 2 (10.0%) | 4 (20.0%) | 1 (8.3%) |
| Anterior wall | 16 (14.7%) | 3 (15.0%) | 2 (10.0%) | 3 (25.0%) |
| Inferior wall | 31 (28.4%) | 0 | 2 (10.0%) | 0 |
| Posterior wall | 1 (0.9%) | 0 | 1 (5.0%) | 0 |
| Apex | 18 (16.5%) | 0 | 2 (10.0%) | 0 |
| Papillary muscle | 1 (0.9%) | 0 | 1 (5.0%) | 0 |
| Summit | 1 (0.9%) | 1 (5.0%) | 0 | 1 (8.3%) |
| Interventricular septum | 19 (17.4%) | 2 (10.0%) | 4 (20.0%) | 1 (8.3%) |
| Right ventricular |  |  |  |  |
| Free wall | 1 (0.9%) | 2 (10.0%) | 1 (5.0%) | 0 |
| Inferior wall | 0 | 1 (5.0%) | 0 | 0 |
| RVOT | 0 | 1 (5.0%) | 0 | 0 |
| Interventricular septum | 3 (2.8%) | 4 (20.0%) | 1 (5.0%) | 3 (25.0%) |
| Papillary muscle | 1 (0.9%) | 0 | 0 | 0 |
| AMC | 0 | 1 (5.0%) | 0 | 1 (8.3%) |
| Epicardium | 5 (4.6%) | 3 (15%) | 2 (10.0%) | 2 (16.7%) |

AMC, Aortic-mitral continuity; RVOT, right ventricular outflow tract; VT, ventricular tachycardia.
